# Associations of Suicide Risk and Community Integration Among Patients With Treatment-Resistant Depression

**DOI:** 10.3389/fpsyt.2022.806291

**Published:** 2022-03-02

**Authors:** Pham Thi Thu Huong, Chia-Yi Wu, Ming-Been Lee, I-Ming Chen

**Affiliations:** ^1^School of Nursing, National Taiwan University College of Medicine, Taipei, Taiwan; ^2^Faculty of Nursing and Midwifery, Hanoi Medical University, Hanoi, Vietnam; ^3^National Institute of Mental Health, Bach Mai Hospital, Hanoi, Vietnam; ^4^Department of Nursing, National Taiwan University Hospital, Taipei, Taiwan; ^5^Taiwanese Society of Suicidology and Taiwan Suicide Prevention Center, Taipei, Taiwan; ^6^Department of Psychiatry, National Taiwan University Hospital, Taipei, Taiwan; ^7^Department of Psychiatry, Shin Kong Wu Ho Su Memorial Hospital, Taipei, Taiwan

**Keywords:** treatment-resistant depression, suicidal ideation, community integration (MeSH), gender differences, recovery

## Abstract

**Introduction:**

Treatment-resistant depression (TRD) is one of the primary causes of disability and a major risk for suicide among patients living in the community. However, the suicide risks and care needs for safety among patients with TRD during the community reintegration process appear to be underestimated. This study aimed to investigate the association between community integration and suicide risks among patients with treatment-resistant depression (TRD) with sub-analysis by gender.

**Methods:**

Patients diagnosed with major depressive disorder were recruited upon psychiatrists' referral in two general hospitals in northern Taiwan during 2018–2019. The participants who experienced more than two failed treatments of antidepressants with partial remission were defined as TRD. A structured questionnaire was used to collect socio-demographic, suicidality, and psychosocial information.

**Results:**

In a total of 125 participants, gender difference was identified in certain community integration aspects such as home integration, productivity, and electronic social networking. The male participants appeared to have better involvement in social contact with internet but slightly less video link than women, while women had higher level of home integration in the past month. The participants who performed worse in the social integration and better home-based activity or productivity levels had higher suicide risks including suicide ideation and overall suicide risks.

**Conclusions:**

Community integration levels of home, social, and productivity were associated with suicidality in terms of overall suicide risk and recent suicide ideation. Facilitation of community integration at home and life arrangements might reduce suicide risks in TRD patients.

## Introduction

Major depressive disorder (MDD) is the leading cause of disability. It is featured by its chronicity, recurrence, gender differences, and high-risk for suicide (1, 2). Approximately half of MDD patients would respond to an initial trial of an antidepressant, with only a third reaching clinical remission (3). Despite the varied definitions across the world, TRD has been defined and recognized as not responding to two adequate antidepressant medication regimens for patients with MDD (4). Further, two review and consensus studies of the Asian populations defined TRD as failure of at least two antidepressant trials at an adequate dose for 6–8 weeks during any MDD episode (5, 6). The management of disease and follow-up care among patients with TRD is put forward as an important issue.

More than 90% of people with completed suicide have been linked to a mental disorder (7), with MDD being the most common disease related to suicide (8, 9). Furthermore, history of suicide attempts and comorbid psychiatric disorders were the strongest risk factors for future attempts of those with TRD (10). Researchers identified the prevalence of suicide ideation among patients with TRD was 2-fold higher than in the non-TRD group (11). Apart from suicide risks, TRD patients had significantly lower productivity loss and activity impairment as compared to non-TRD and the general population (11). Moreover, a systematic review found that patients who admitted with suicidal thoughts or behaviors had near 200 times more risk of global suicide rate, even many years after discharge; in contrast, patients with previous psychiatric hospitalization have 30 times higher rates of suicide (12). Despite the continuous advancement in neuroscience and cutting-edge role of ketamine and ketamine treatment (9, 13) as well as in the knowledge of human behavior pathophysiology, currently, suicidal behavior represents a puzzling challenge. The complexity of suicidality in MDD has been explained by various possible pathophysiological factors as the role of neurobiological, neuroimmunological biomarkers, brain-derived neurotrophic factor, and other neuromodulators (9). However, the need of careful assessment and patient follow-ups with high risk of suicide in the community is less researched yet composed a critical part of suicide prevention in modern psychiatry (9).

Research in Taiwan indicated that during the trajectory of community life recovery, patients with TRD had difficulties integrating to social life due to multifactorial barriers including poor personal conditions and social adaptation skills (14). In this context, researchers in previous studies have introduced the concept of community reintegration for better recovery in TRD, including family life, productivity, social activities, and electronic social networks. A recent study suggested that well-integrated life in community was a protective factor associating with current suicide ideation in the past 2 weeks (15). Similarly, two prospective cohort studies addressed the negative correlation between suicide and social integration among both genders in the general population in the USA (16, 17). Although researchers excluded participants with poor mental health status, positive social integration was a potential protective factor for 2- and 3-fold reduction of suicide risk in men and women, respectively. However, the evidence about the correlation between community integration and suicide risks was limited.

The study sought to examine whether community integration level was associated with suicidality among a cohort of TRD patients in northern Taiwan, with specific interest in exploring the gender differences in community integration features.

## Methods

### Design

This cross-sectional study was nested in a 3-year prospective study investigating the psychosocial characteristics and non-pharmacological management for Taiwanese patients with TRD (Protocol ID: 201612198RINB). A cohort of TRD was established in 2018, with nurse-led group intervention and long-term follow-ups in the following 2 years. The current study analyzed the associations of suicide risks and the community integration levels of the TRD cohort.

### Participants

The study participants were referred by psychiatrists at two general hospitals in northern Taiwan during January 2018 to October 2019. The sources of referral included psychiatric day-care service, outpatient department, or inpatient units. They were all confirmed with the diagnosis of MDD according to the 5th version of the Diagnostic and Statistical Manual and remitted partially in the recovery process for more than 2 years before participation in this study. All the patients were under medication treatment at the time of recruitment, albeit the existence of psychological or other therapeutic treatments. The inclusion criteria was an experience of two types of antidepressants in a recent episode of MDD according to the medical records. The exclusion criteria included cognitive impairment, communication barriers, and unstable disease conditions.

### Data Collection

Before formal data collection, two psychiatrists of the research team underwent discussions of recruitment definition and procedures in two consensus meetings. The study was approved by two Ethics Committees in the study hospitals (201612198RINB and 20190106R). All the participants agreed to take part and signed the informed consent. A structured interview with questionnaire was conducted with each patient by the assistant and double-checked by the corresponding author.

### Measurements

The measurements included socio-demographic and psychosocial information and the assessments toward suicidality and community integration status. Suicidality was defined as different suicide risk factors including the overall suicide risk, suicide ideation and suicide attempt in a week or lifetime.

#### The Socio-Demographic and Psychosocial Information

Participants' socio-demography was collected by variables such as gender, age, education years and level (below junior high/senior high/above university), marital status (single/married or cohabited/others), occupation status (yes/no), religious belief (yes/no), and status of living alone (yes/no). Further, frequent feelings of hopelessness were inquired and recorded as yes/no.

#### Suicidality Measures

The term “suicidality” in this study was defined as including suicide ideation and suicide attempt. Two major scales were adopted to the assessment of suicidality, the five-item Brief Symptom Rating Scale (BSRS-5) and the nine-item Concise Mental Health Checklist (CMHC-9). The BSRS-5 was developed to identify recent psychiatric morbidity and suicide ideation in community subjects and psychiatric patients (18, 19). The scale contained the following five items of psychopathology and an additional item assessing suicide ideation: (1) having trouble falling asleep (insomnia); (2) feeling tense or keyed up (anxiety); (3) feeling easily annoyed or irritated (hostility); (4) feeling low in mood (depression); (5) feeling inferior to others (interpersonal hypersensitivity: inferiority). The additional item inquired, “Do you have suicide ideation in the past week?”. The participants were asked to rate symptoms on a five-point Likert scale from 0, not at all; 1, a little bit; 2, moderately; 3, quite a bit; and 4, extremely. The total score was calculated by computing the first five items, with cut-off points of 5/6, 9/10, and 14/15 standing for mild/moderate/severe levels of mental distress. In this study, the Cronbach's alpha of BSRS-5 was 0.84.

The CMHC-9 was a nine-item short assessment of recent psychological distress and overall suicide risks in the community and clinical settings (20). It is a brief and effective tool for high-risk detection with satisfactory sensitivity (92%) and specificity (82%) in identifying recent suicide ideation. The nine items were divided into two core components of assessment, i.e., psychopathology/mental distress and major suicide risk factors. Each item was rated as 0 (no distress at all) or 1 (significant distress), with the timeframe spanning from past-week (5-item mental distress), lifetime (suicide attempt), and future (suicide intention) in order to objectively assess the overall risk of suicide. The total score ranges from 0 to 9, with the cutoff value over 4 points indicating higher suicide risk. The Cronbach's alpha for the scale in this study was 0.77.

#### The Revised Community Integration Questionnaire (CIQ-R)

The 18-item CIQ-R was used to assess life conditions across four components: home integration (items 1–5), social integration (items 6–11), productivity (items 12–15), and electronic social networking (items 16–18) (21). The original version of Community Integration Questionnaire has been widely utilized to measure community integration following traumatic brain injury and provide further direction for a clinical or research setting (22). It was also expanded to other target population as physical disability, spinal cord injury, brain tumors, and so on (21). The home integration component had scores from 0 to 2 points reflecting a person's autonomy in performing home activities; the score of 2 means mostly done by oneself, 1 means partly self or by family members, and 0 means all by the family. The assessment of social involvement or productivity in the past month was based on performance frequency, dividing into 0 (never), 1 (1–4 times), or 2 (over 5 times). The electronic social networking included the frequency of contact to others using internet, online video link, or text message with scores as every day or most days = 2, almost every week = 1, and Seldom/never = 0. Higher scores of CIQ-R stand for better integration level or social activity involvement in actual or online community settings. The Cronbach's alpha was 0.66 in this study.

### Data Analysis

Statistical calculations were carried out using SPSS 22.0. The means, standard deviations, and frequencies were calculated for quantitative variables; Cronbach's alpha was used to estimate the internal consistency of BSRS-5; CMHC-9, CIQ-R. Independent *t*-tests were adopted to explore the difference in each CIQ-R item between men and women and the difference of each CIQ-R subscale from suicidality variables. To examine a relationship between CIQ-R subscales and overall suicidality variables, Pearson correlation or *t*-test coefficients were obtained and computed. Statistical significance was set at a level of *p* < 0.05.

## Results

### The Socio-Demographic and Suicidality Status

A total of 125 patients with TRD participated in this study. Three-fourth of all participants were women (*n* = 91, 72.8%). The mean age of patients was 55.4 (SD = 14.6) within a range of 20–80 years old. Nearly half of the samples (*n* = 58, 46.4%) had a degree above university with average education of 11.9 years. More than half of the people (*n* = 68, 54.4%) were married or cohabited. Two-thirds reported a religious belief (*n* = 94, 75.2%). Only one in five (*n* = 26, 20.8%) had a full-time job and closely one-third (31.2%) reported unemployment at the time of study. The vast majority of participants lived with at least one cohabitant (*n* = 104, 83.2%). More than half of participants felt hopeless of their future (*n* = 85, 68%). According to the scores of BSRS-5, the patients presented a higher level of mental distress (M = 10.8, SD = 5.4). Nearly all participants seriously considered suicide in their lifetime (*n* = 118, 94.4%), 52 patients (41.6%) revealed recent suicide ideation in the past week, and 70 (56%) had lifetime suicide attempt. Moreover, the severity of suicide ideation during the lifetime was self-rated as 7.2 points in average in a scale of 0–10. The overall suicide risk was high with 4.0 ± 2.6 points assessed by the CMHC-9 (Table 1).

**Table 1:** The socio-demographic and suicidality status of patients with treatment-resistant depression (*N* = 125).

**Variable**		***n*/Mean ±SD (range)**	**%**
Gender	Female	91	72.8
	Male	34	27.2
Age group		55.4 ± 14.6 (20–80)	
	20–44	29	23.2
	45–64	54	43.2
	65–80	42	33.6
Education	Below junior high school	42	33.6
	Senior high school	25	20.0
	Above university	58	46.4
Education years		11.9 ± 4.8 (0–20)	
Marital status	Single	26	20.8
	Married/Cohabited	68	54.4
	Divorced/Separated	18	14.4
	Widowed	13	10.4
Religion	No	31	24.8
	Yes	94	75.2
Job	None	39	31.2
	Yes	86	68.8
Living alone	Yes	21	16.8
	No	104	83.2
Feeling hopeless	Yes	85	68.0
	No	40	32.0
Mental distress (BSRS-5)^*^	Total score	10.8 ± 5.4 (0–20)	
	Insomnia	99	79.2
	Anxiety	106	84.8
	Irritability	102	81.6
	Depression	108	86.4
	Inferiority	96	76.8
Suicide ideation (1 week)	Yes	52	41.6
	No	73	58.4
Lifetime suicide ideation	Yes	118	94.4
	No	7	5.6
Lifetime suicide attempt	Yes	70	56.0
	No	55	44.0
Overall suicide risk (CMHC-9)^∧^	Total score	4.0 ± 2.6 (0–9)	

* *Each item in the BSRS-5 was scored ≥1 point in the 0–4 Likert scale*.

∧ *CMHC-9: The nine-item Concise Mental Health Checklist*.

### The Association Between Psychosocial Factors, Suicidality, and Community Integration

In this study, suicidality was defined to represent the overall risk that includes both suicide ideation and suicide attempt. This section analyzed whether higher suicidality would be associated with community integration levels in terms of home, social, productivity, and electronic contact components. As seen in Table 2, patients with lifetime suicide ideation showed higher level of productivity (*t* = −4.48, *p* < 0.001). The significant correlation between home integration and social integration to the overall suicide risk score (*p* < 0.05) was also identified. This indicated that people who engaged more actively in home-based activities such as housework were more likely to demonstrate a higher rate of overall suicide risk. Specifically, those who had higher social integration scores were significantly associated with lower overall suicide risk (*p* < 0.01). Moreover, there was a significant difference in the mean score of social integration between patients who reported recent suicide ideation (M = 5.0, SD = 2.1) compared to those without such ideation (M = 6.0, SD = 2.3) (*t* = 2.61, *p* < 0.05). That is, patients with recent suicide ideation performed worse in the social integration component than those without such ideation. These observations might be explained by the characteristics of TRD and are discussed further in the discussion section.

**Table 2:** The associations between community integration conditions and suicidality (*N* = 125).

**Mean (SD)**	**Overall suicide risks (*r* value)**	**Past-week suicide ideation**		**t**	**Lifetime suicide ideation**		**t**	**Lifetime suicide attempt**		**t**
		**Yes**	**No**		**Yes**	**No**		**Yes**	**No**	
Home integration	0.19*	5.6 (3.8)	5.3 (3.3)	−0.54	5.4 (3.5)	6.6 (3.2)	0.88	5.7 (3.8)	5.1 (3.2)	−0.99
Social integration	−0.30**	5.0 (2.1)	6.0 (2.3)	2.61*	5.5 (2.3)	6.0 (1.4)	0.56	5.2 (2.2)	5.9 (2.2)	1.76
Productivity	0.11	3.0 (2.0)	2.6 (1.8)	−1.11	2.8 (1.9)	2.0 (0.0)	−4.48***	3.0 (1.9)	2.5 (1.8)	−1.57
Electronic Social networking	0.03	2.4 (1.8)	2.2 (1.8)	−0.55	2.3 (1.8)	1.4 (1.1)	−1.28	2.3 (1.8)	2.1 (1.7)	−0.68
CIQ-R total	0.05	17.1 (5.8)	17.1 (5.4)	0.01	17.2 (5.6)	17.7 (4.9)	0.26	17.6 (5.9)	16.7 (5.0)	−0.85

*p < 0.05; **p < 0.01; ***p < 0.001. t/r: The values were derived from Independent t-tests or Pearson correlations.

*SD, standard deviation*.

### Gender Differences in Community Integration

Table 3 shows the gender differences in community integration features. Although male participants were much less than their female counterparts, the analysis revealed the current situation of female predominance in service-seeking behavior for depression. Gender inequality was found to be significant in specific items as preparing meals, housework, childcare, current work situation, and social contact using the internet by writing or video call (*p* < 0.05). For the specific question, “Who usually prepares meals in your household?”, while a half of women (50.5%) prepared meals alone, around two-thirds of men (70.6%) had someone prepare for them (*p* < 0.001).

**Table 3:** The descriptions of Community Integration Questionnaire-Revised items (*N* = 125).

**Items**		***n* (%)**	**Male (*n* = 34)**	**Female (*n* = 91)**	**χ^2^**
**Home integration (item 1–6)**					
1	Buying groceries	Yourself alone	9 (26.5)	37 (40.7)	1.37
		Yourself and someone else	12 (35.3)	27 (29.7)	
		Someone else	13 (38.2)	27 (29.7)	
2	Prepares meals	Yourself alone	5 (14.7)	46 (50.5)	4.79***
		Yourself and someone else	5 (14.7)	17 (18.7)	
		Someone else	24 (70.6)	28 (30.8)	
3	Housework	Yourself alone	3 (8.8)	45 (49.5)	4.75***
		Yourself and someone else	11 (32.4)	19 (20.9)	
		Someone else	20 (58.8)	27 (29.7)	
4	Child care	Yourself alone	0 (0)	8 (8.8)	3.12**
		Yourself and someone else	4 (11.8)	16 (17.6)	
		Someone else	10 (29.4)	18 (19.8)	
5	Social arrangements	Yourself alone	3 (8.8)	9 (9.9)	−1.30
		Yourself and someone else	11(32.4)	12 (13.2)	
		Someone else	20 (58.8)	70 (76.9)	
6	Financing	Yourself alone	19 (55.9)	43 (47.3)	−0.63
		Yourself and someone else	3 (8.8)	13 (14.3)	
		Someone else	12 (35.3)	35 (38.5)	
**Social integration (item 7–11)**					
7	Shopping	Never	3 (8.8)	18 (19.8)	−0.43
		1–4 times	18 (52.9)	34 (37.4)	
		5 or more	13 (38.2)	39 (42.9)	
8	Leisure activities	Never	5 (14.7)	23 (25.3)	−1.52
		1–4 times	14 (41.2)	39 (42.9)	
		5 or more	15 (44.1)	29 (31.9)	
9	Visiting friends/relatives	Never	20 (58.8)	56 (61.5)	0.24
		1–4 times	14 (41.2)	30 (33.0)	
		5 or more	0 (0)	5 (5.5)	
10	Companies when doing leisure activities	Mostly alone	10 (29.4)	19 (20.9)	0.59
		Mostly with friend	1 (2.9)	9 (9.9)	
		Mostly with family members	23 (67.6)	63 (69.2)	
11	Having a best friend with whom you confide	Yes	21 (61.8)	58 (63.7)	0.20
		No	13 (38.2)	33 (36.3)	
**Productivity (item 12–15)**					
12	Traveling outside	almost every day	28 (82.4)	69 (75.8)	−0.79
		almost every week	3 (8.8)	10 (11.0)	
		seldom/never	3 (8.8)	12 (13.2)	
13–15	Current work situation∧	0	18 (52.9)	67 (73.6)	−2.16*
		1	1 (2.9)	2 (2.2)	
		2	1 (2.9)	5 (5.5)	
		3	3 (8.8)	8 (8.8)	
		4	11 (32.4)	6 (6.6)	
		5	0 (0)	3 (3.3)	
**Electronic social networking (item 16–18)**					
16	Frequency of writing to people using the Internet	Everyday/most days	22 (64.7)	29 (31.9)	−3.35**
		Almost every week	2 (5.9)	9 (9.9)	
		Seldom/never	10 (29.4)	53 (58.2)	
17	Frequency of social contact with video link	Everyday/most days	0 (0)	7 (7.9)	1.10*
		Almost every week	3 (8.8)	8 (9.0)	
		Seldom/never	31 (91.2)	74 (83.1)	
18	Frequency of talking/messaging by phone	Everyday/most days	21 (61.8)	37 (41.6)	−1.74
		Almost every week	5 (14.7)	22 (24.7)	
		Seldom/never	8 (23.5)	30 (33.7)	

*p ≤ 0.05 **p < 0.01 ***p < 0.001; t: The values were derived from independent t-tests. Scoring of each item was based on degree of autonomy or frequency, with higher score indicates more independence or social contact.

*∧Current working status: 0 (Not working, not looking for work, not going to school, no volunteer activities), 1 (Volunteer 1–4 times and not working, not looking for work, not in school), 2 (Actively looking for work and/or volunteers 5 or more times), 3 (Attends school part-time or works part-time), 4 (Attends school full-time or work full-time), 5 (Works full-time and attends school part-time or attends school full-time and works part-time)*.

In the domain of home integration, male and female participants showed remarkably different performance in their reports. In the item, “In your home, who usually does normal everyday housework?”, nearly half (49.5%) of women were active in doing normal everyday housework alone. On the other hand, only 8.8% men did the house work (*p* < 0.001). With patients who had children, we assessed, “Who usually cares for the children in your home?”. Not any male participant took care of their children alone and only 11.8% (*n* = 4) of male patients could take care of their children together with another person. In contrast, 8.8% (*n* = 8) of women usually cared for their children alone and 17.6% (*n* = 16) could look after their children with another person (*p* < 0.01).

In the aspect of current work situation, two-thirds of women (*n* = 67, 73.6%) and half of men (*n* = 18, 52.9%) had neither job, learning, nor volunteer activities. Specifically, one-third of men (*n* = 11, 32.4%) attended school or worked full-time, but only 6.6% (*n* = 6) of women did so. Only 3% of women reached the highest level of current work situation; however, no male participant reported the same level (*p* < 0.05). Nearly five times of men were under employment or school learning as compared to women (32.4% vs. 6.6%), indicating the lower work conditions for women with TRD.

In addition, gender difference was found significant in the frequency of writing to people using the internet and using a video link for social contact (*p* < 0.05). Two-thirds of men (64.7%) wrote for social contact via the internet every day or most of the days, whereas less than one-thirds of women (31.9%) did. More than half of women (58.2%) seldom wrote to people for social contact. Finally, while no men reported using a video link for social contact every day, 7.9% women did so (*p* < 0.05).

## Discussion

Our findings highlighted that over ninety percent of patients with TRD seriously considered suicide in their lifetime and nearly half either revealed recent suicide ideation (in the past week) or history of self-harm/suicide attempts. Moreover, significant differences were identified in the levels of four components of the community integration assessment between male and female groups. For instance, male participants appeared to have better involvement in social contact with internet but slightly less video link than women; and women had higher level of home integration. In addition, TRD patients who had lower scores in the social integration and higher functioning in home integration were more likely to demonstrate higher scores in overall suicide risks. Those with TRD and recent suicide ideation were involved less in activities of social integration than those without recent ideation. Intriguingly, patients with lifetime suicide ideation presented significantly higher scores of productivities than those without that experience.

This study applied the CIQ-R to assess community life features in the recovery progress of patients with TRD. The issue was rarely investigated in the past when most prior evidence about TRD in Taiwan focused on epidemiology (23), pharmacological treatment (24, 25), and complementary treatment as transcranial magnetic stimulation or prefrontal theta-burst stimulation (26, 27). A previous study displayed long-term effects of functional impairment; severe depressive patients had higher rates of suicide (15) and higher all-cause mortality compared with non-TRD (28, 29). Whereas, in the present study, the majority of individuals with TRD presented high levels of mental distress which was assessed by the BSRS-5. Similar to previous study that compared to general population, psychiatric patients appeared to be 4.6–6.7-times more severe in psychological distress (14), and ~ 7.2-fold more severe for patients with TRD in this study. The results offer an evidence that chronic psychological distress is a significant concern that needs more attention for patients with TRD.

Gender difference of depression was found to be interpreted as related to presentation and precipitation of the disorder (30). Gender plays a role in community integration process of TRD, as evident in this study. Among 125 patients of our study, the number of women was 2.7-fold higher than men, which is consistent in ratio with other studies in Taiwan (23, 24), China (31), United States (27), and Sweden (10). Regarding the gender issue, our study found significant differences in specific items of the community integration scale, such as preparing meals, housework, childcare, current work situation, and social contact using the internet by writing or video call. Women demonstrated significantly higher scores in home integration scale (preparing meals, housework, and childcare) than men, consistent with other reports (32, 33). However, this could be explained by the fact that Taiwanese women are more likely to take care of household chores whether they are in good health or not. Appealingly, it was found that patients who had higher functioning in the home integration domain showed higher score in the overall suicide risks. Of interest, various factors effected low involvement in home activities, including lack of necessity and motivation (32). One justifiable reason could be that even under severe depression and suicide risk, women with TRD still take responsibilities of house work in the society of Taiwan. These results suggest that in addition to providing different approaches in life skills training, healthcare providers could focus on reinforcement of a patient's motivation as well as promote the family members' shared responsibilities with the patients. Furthermore, mental health professionals should enhance sufficient knowledge in psychoeducation intervention and emotional support toward patients with TRD and their caregivers in order to decrease the conflict caused by and the burden from the family.

In the present study, low social integration was found to be significantly associated with higher level of overall suicide risk. Of interest, patients with recent suicide ideation got lower average scores in the domain of social integration (i.e., less frequent shopping, leisure activities, visiting friends/relatives, or hardly any confiding relationship) than those with no recent suicide ideation. Interestingly, these are entirely related to the concept of connectedness which has been demonstrated in various empirical studies regarding powerful protective factors to prevent suicide behavior (34, 35). The term social integration encompasses concepts such as social networks, social support, and social engagement, which were found to be positively associated with psychological integration in the communities during the pathway to recovery (36). Similar to the fact that well-integration in the community reflected lower risk in suicide ideation, being alone and isolated were also evident in predicting the remission rate (1, 29), disability (37), and suicide attempts (10). The positive outcomes of friendship support for patient with serious mental illness were identified in studies related to social relationships (38). In contrast to friendship cooperation that requires both directions and reciprocity, the family members and healthcare providers are more likely to be one-sided in support for depressive patients. This robust evidence highlights the importance of social relationships as a target strategy for mental health staff to promote the importance of maintaining social connections of TRD patients for better quality of life or suicide prevention. In addition to providing enough information in psychotherapy programs and emphasizing the value of a healthy social relationship (39), healthcare providers can help the patients be aware and recognize harmful relationships which may worsen mental health development and deteriorate interpersonal relationship during the recovery process. Our finding lends support to suicide prevention guidelines in clinical settings regarding the importance of screening social integration level for suicide risk assessment (17).

Lastly, it was found that patients with lifetime suicide ideation responded with higher average scores in the productivity component (e.g., frequency of traveling outside the home and current performance status related to school, work, and volunteer activities). There was evidence that patients with suicide ideation appeared to have greater level of perfectionism and work performance compared to patients without suicidal ideation (40). Perfectionism is a multidimensional personality trait characterized by exceeding high personal performance standards by overly self-critical evaluation (41). Individuals with perfectionism attempt to perform perfectly to meet the social expectation, yet they may live with fear of mistake and self-criticism. Hence, the most frequently cited relationship with perfectionism was major depression, anxiety, obsessive-compulsive disorder, and suicidality in the previous studies. Therefore, our findings could support the fact that patients with TRD are struggling in work/life performances related to their social roles during the recovery process. According to Davidson and Roe (42) recovery is considered when a person is able to have a meaningful life while continuing to have a mental illness. That is a suggestion for mental health providers to objectively assess and understand the ability, current needs, and personal characteristics of patients with TRD in order to identify facilitators for community reintegration and barriers to illness management during depression treatment. Finally, to reduce suicidality and build up better recovery for those with TRD, mental health policy should maintain a safe and supportive community environment for patients with TRD to adjust their life roles to adapt better in the community.

In interpreting these results, several limitations should be taken into consideration. First, the study simply analyzed cross-sectional data with a small sample size; selection bias and reverse causality might limit the generalizability of the findings. In addition, one-third of our sample was around 65–80 years old, which could affect to the level of community integration due to their functional impairment of TRD. Future studies are suggested to apply longitudinal designs in identifying the causal relationship between community integration and suicide risks. Finally, non-pharmacological intervention studies for patients with TRD may guide future development of better care quality in the trajectory of community recovery.

## Conclusion

This was to our knowledge the first study which examined the association between community integration and suicidality among patients with TRD. Our results revealed a high suicide ideation and attempt rate among this patient group. It also highlighted that the community integration levels of home, social, and productivity were associated with overall suicide risk and recent suicide ideation with gender differences in an Asian context. Facilitation of community integration at home and life arrangements might reduce suicide risks in TRD patients. In conclusion, in order to enhance the quality of community life and mental health care for patients with TRD during recovery process, healthcare providers should actively empower their community involvement, social adaptation skills, as well as self-care needs between genders in the chronic illness trajectory.

## Data Availability Statement

The raw data supporting the conclusions of this article will be made available by the authors, without undue reservation.

## Ethics Statement

The studies involving human participants were reviewed and approved by the Ethics Committee of the National Taiwan University Hospital. The patients/participants provided their written informed consent to participate in this study.

## Author Contributions

PH: data curation, formal analysis, and writing—original draft. C-YW: conceptualization, data curation, formal analysis, investigation, methodology, supervision, and writing—review and editing. M-BL: investigation, conceptualization, methodology, supervision, writing-original draft, and writing—review and editing. I-MC: conceptualization, supervision, and writing–review and editing. All authors have read and approved the final manuscript.

## Funding

This project was funded by the Taiwan Ministry of Science and Technology (MOST106-2314-B-002-010-MY3). The role of the sponsor did not affect the research team in the following aspects: study design, data collection, analysis, interpretation of data, writing of the report, and decision to submit the articles for publication.

## Conflict of Interest

The authors declare that the research was conducted in the absence of any commercial or financial relationships that could be construed as a potential conflict of interest.

## Publisher's Note

All claims expressed in this article are solely those of the authors and do not necessarily represent those of their affiliated organizations, or those of the publisher, the editors and the reviewers. Any product that may be evaluated in this article, or claim that may be made by its manufacturer, is not guaranteed or endorsed by the publisher.
